# New Pharmaceutical Salts of Trazodone

**DOI:** 10.3390/molecules26030769

**Published:** 2021-02-02

**Authors:** Jolanta Jaśkowska, Przemysław Zaręba, Anna Drabczyk, Agnieszka Kozak, Izabela D. Madura, Zbigniew Majka, Edyta Pindelska

**Affiliations:** 1 Institute of Organic Chemistry and Technology, Faculty of Chemical and Engineering and Technology, Cracow University of Technology, 24 Warszawska Street, 31-155 Cracow, Poland; przemyslaw.zareba@pk.edu.pl (P.Z.); anna.drabczyk@pk.edu.pl (A.D.); 2 Department of Analytical Chemistry and Biomaterials, Faculty of Pharmacy, Medical University of Warsaw, Banacha 1, 02-093 Warsaw, Poland; asokal@wum.edu.pl; 3 Faculty of Chemistry, Warsaw University of Technology, Noakowskiego 3, 00-664 Warsaw, Poland; izabela@ch.pw.edu.pl; 4 Zbigniew Majka Consulting, ul Górczewska 200c /58, 01-460 Warszawa, Poland; zbig_majka@wp.pl

**Keywords:** trazodone, drug design, dissolution, crystal structure, solid-state NMR (SSNMR) spectroscopy, GIPAW calculation

## Abstract

New pharmaceutically acceptable salts of trazodone (trazodone hydrogen bromide and trazodone 1-hydroxy-2-naphthonic acid) for the treatment of central nervous system disorders are synthesized and described. Although trazodone salts are poorly crystalline, single-crystal X-ray diffraction data for trazodone 1-hydroxy-2-naphthonic acid were collected and analyzed as well as compared to the previously described crystal structure of commercially available trazodone hydrochloride. The powder samples of all new salts were characterized by Fourier transform infrared spectroscopy, X-ray diffraction and ^13^C solid-state nuclear magnetic resonance spectroscopy. Spectroscopic studies were supported by gauge including projector augmented wave (GIPAW) calculations of carbon chemical shielding constants. The main goal of our research was to find salts with better physicochemical properties and to make an attempt to associate them with both the anion structure and the most prominent interactions exhibited by the protonated trazodone cation. The dissolution profiles of trazodone from tablets prepared from various salts with lactose monohydrate were investigated. The studies revealed that salts with simple anions show a fast release of the drug while the presence of more complex anion, more strongly interacting with the cation, effects a slow-release profile of the active substance and can be used for the preparation of the tables with a delay or prolonged mode of action.

## 1. Introduction

2-{3-[4-(3-Chlorophenyl)-piperazin-1-yl]-propyl}-[1,2,4]-triazolo-[4,3-a]-pyridin-3-(2*H*)-one, known as “trazodone”, is used to treat mood disorders, such as depression [1]. Trazodone belongs to the group of drugs that block serotonin receptors and inhibit the reuptake of serotonin (SARI) [2]. It was introduced into medicine in the early 1970s, and in the 1980s it was the most frequently prescribed antidepressant in the United States [3,4]. The subsequent introduction and promotion of drugs inhibiting serotonin reuptake (SSRI) resulted in a decrease in interest in antidepressants with a different mechanism of action, but the analysis of the pharmacological profile of trazodone allowed for the extension of therapeutic indications and recognition of its many possibilities. Today, this drug has once again found a prominent place in the pharmacological treatment of depression. One of the new, very promising additional indications is the possibility of using trazodone in patients with neuropathic pain in the course of neoplastic disease [5]. Trazodone is an antagonist of 5-HT_2A_ and 5-HT_2C_ receptors and an inhibitor of the serotonin transporter (SERT). Besides, it blocks adrenergic receptors—α_1_ and α_2_ and histamine H_1_. Unlike other antidepressants, trazodone has a weak effect on transporters of other amines: dopamine (DAT) or noradrenaline (NET) [6]. In the case of trazodone, the therapeutic dose is 75–100 mg daily in a single dose, the dose can be increased to 300 mg daily in divided doses. In justified cases, the dose may be increased to 600 mg per day.

There are several methods of obtaining trazodone known, yet recently a new method via the reaction of 2-(3-halopropyl)-1,2,4-triazolo-[4,3-a]-pyridin-3-(2*H*)-one and 1-(3-chlorophenyl) piperazine hydrochloride under microwave radiation in the presence of potassium carbonate has been developed [7]. However, to search for more effective methods of pharmacotherapy, not only new medicinal substances are sought, but also new forms of administration of already known drugs. Modifying the form of a drug may change the pharmacokinetics of release, and thus a sustained-release (SR) or controlled-release (CR) dosage can be obtained. It is important to underline that the drug substances with the altered release time must be well known and must have a defined relationship between a dose and its therapeutic action.

More than 80% of all dosage forms of drugs are administered orally. However, it carries some barriers related to absorption, e.g., extreme fluctuating pH of the stomach, the activity of enzymatic systems, the permeability of mucus covering the gastrointestinal tract [8,9]. Additionally, the physiological conditions in the gastrointestinal tract, such as variable passage speed or the amount of fluid, can significantly affect bioavailability [10]. Hence, modified-release drug forms are being developed to limit the influence of physiological factors and to have greater control over bioavailability, and thus also limit fluctuations in blood concentration of the administered substance [8,9]. For the orally administered drugs, retarded forms are often desirable. They can provide increased protection of the gastrointestinal tract against the harmful effects of the active substance or improve bioavailability by releasing the drug at a specific site in the gastrointestinal tract [10,11,12,13]. Moreover, with appropriately modified drugs it is also possible to obtain very good therapeutic effects, often with reduced therapeutic doses of active substances and, at the same time, the limitation of side effects [11].

**Figure 1:**
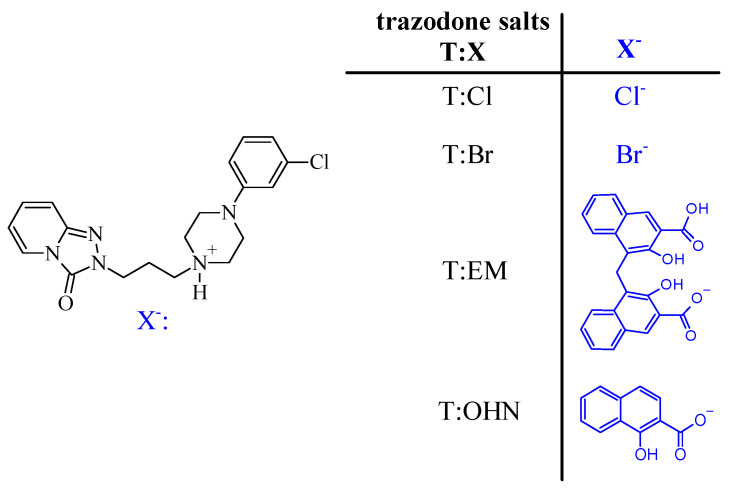
The studied trazodone salts: hydrochloride (T:HCl), hydrobromide (T:HBr), 1-hydroxy-2-naphthonic acid (T:OHN) and embonate (T:EM).

In pharmaceutical practice, only trazodone hydrochloride (T:HCl) is commercially produced (e.g., Trazodone Neuraxpharm (Trazodone Glenmark)-Neuraxpharm, Trittico^®^ CR or Trittico^®^ XR-Angelini). Although synthesis of several trazodone salts has been described in the literature (e.g., sulfate, succinate, maleate, *p*-toluene sulfonate [14], benzoate [15], acetate, propionate, methanesulfonate, ethanesulfonate, benzeneacetate, phosphate, decanoate, oleate, citrate, tartrate, embonate [16], benzilate [17], 2,4-dinitrophenolate [18], nitrate, thiocyanate, tetrafluoroborate [19], iodide, oxalate [20]) the systematic studies connecting the pharmaceutical properties such as dissolution profiles from tablets with the anion structure have never been performed. Herein, we present the dissolution profiles of four trazadone salts from ready-to-sell tablets with lactose (LA). The selected salts are the two newly obtained trazadone hydrobromide (T:HBr) and 1-hydroxy-2-naphthonic acid (T:OHN) as well as trazadone embonic acid (T:EM) and hydrochloride (T:HCl) (Figure 1). These studies are followed by comparative structural analysis of T:OHN and T:HCl crystals to reveal if the anion-cation interactions influence the time required for drug dissolution.

## 2. Results and Discussion

Trazodone was obtained in a solvent-free method of synthesis in the field of microwave radiation under PTC (Phase Transfer Catalysis) conditions [7,21]. The salts were obtained in a reaction with a hydrobromic acid (2 methods) and 1-hydroxy-2-naphthoic acid (3 methods). The T:HBr salt synthesis was carried out in acetone or methanol, then the product was crystallized from ethanol to yield 46–86% yield. The T:OHN salt was obtained by the reaction in acetone, methanol, or ethyl acetate, the product was obtained in a 31–57% yield and did not require additional crystallization [22].

Among all pharmaceutical dosage forms, tablets represent the most popular API delivery system, exhibiting numerous advantages compared to other drug forms. However, only some drugs can be tableted directly without the addition of any excipients, mostly due to diversified active pharmaceutical ingredients (API) stability, which can change, for example upon pressure. On the other hand, the addition of even a small amount of excipients may also change the stability or solubility of the active substances in tablets. In the case of the tested trazodone compounds, we were unable to prepare pure API tablets. During the compression process, the API melted or transformed into another, unstable polymorphic form with a color change. To compare the properties of different trazodone salts from tablets, we prepared tablets with the addition of lactose monohydrate. It is a frequently used excipient, often significantly improving the quality of tablets obtained, as it was also observed with the tablets we prepared (the amount of trazodone in the tablet was set to 75 mg, according to the minimum dose of the drug in the already marketed products, e.g., Trittico CR^®^).

The first factor we decided to measure was the time for a tablet disintegration (Table 1). The disintegration is an important process, which influences a drug bioavailability and ensures its expected concentration in the blood in a given time. In the case of tested trazodone compounds the fastest disintegration was observed for the tablets with T:HBr. This time is 2.5 shorter than the disintegration time of the reference T:HCl tablets. To obtain the complete tablet disintegration for T:OHN and T:EM 24 h were need, and in the case of T:EM the release of only 90% of the API was achieved.

**Figure 2:**
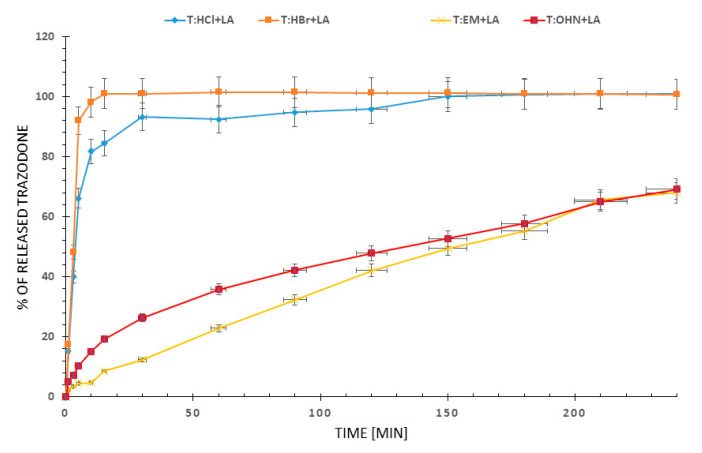
Dissolution profiles in 0.01 M HCl from tablets of studied trazodone salts with lactose monohydrate. The 5% error bars are shown.

To fully compare the properties of the newly obtained stats we evaluated the dissolution profiles of trazodone from tablets containing studied trazodone compounds with lactose monohydrate (LA) addition. Profiles of the trazodone release from T:HCl + LA, T:HBr + LA, T:OHN + LA, T:EM + LA tablets are shown in Figure 2.

The maximum trazodone release in 0.01 M HCl from reference T:HCl + LA tablets is achieved in the first two hours, whereas for all other compounds different time was needed—about 10 min for T:HBr + LA and more than 4 h for T:EM + LA and T:OHN + LA. The best dissolution profile from the pharmaceutical point of view has the T:HBr + LA tablet. In this case, 100% of the API is released during the first 10 min, so a fast mode of action may be assumed. In contrast, in the case of T:EM and T:OHN, the slow dissolution of the drug can be an advantage in the manufacture of prolonged-release tablets. From a therapeutic perspective, this retarded release might be very promising in curing the depression, presumably with a lower or less frequent dosage than usually taken by a patient [11].

One may notice that the dissolution profiles of simple anions (chloride and bromide) differ substantially from the profiles obtained for bigger anions (1-hydroxy-2-naphthoate and embonate). Hence, it might be interesting to compare the trazodone cation structure and its interactions in crystals of these salts and relate it to the dissolution properties. Below, we present the scrutinized analysis of crystal structures for the candidates for tablets of fast (chloride) and slow (1-hydroxy-2-naphthoate) modes of action. It should be emphasized that trazodone salts are weakly crystalline, as evidenced by a small number of structures deposited in the Cambridge Structural Database (CSD) [23]. So far, only seven salts have been structurally characterized [18,19,20]. The crystal structure of T:HCl is deposited in the CSD under the refcode CPTAZP [24]. Although there was no need to obtain a single crystal for X-ray experiments the data have to be improved by adding the positions of hydrogen atoms relative to the position of heavy atoms [25].

**Figure 3:**
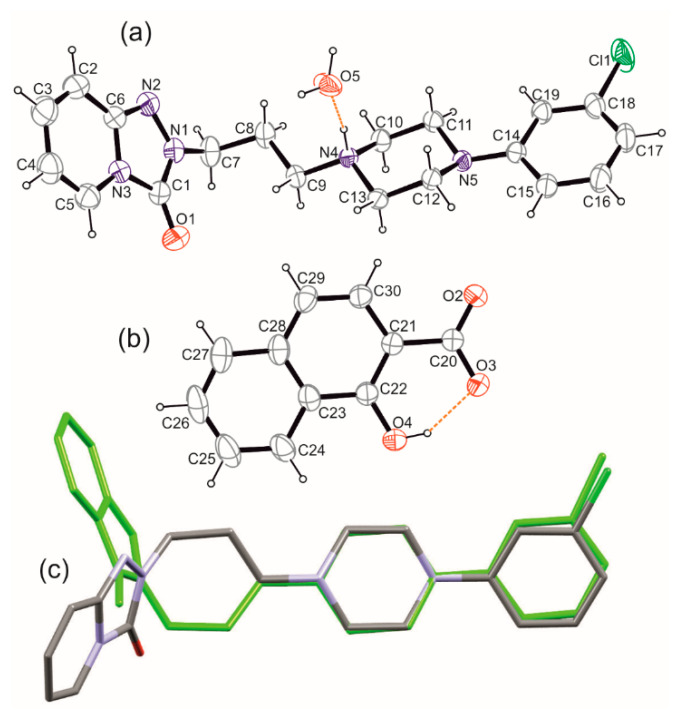
Ortep [26] drawing with the atom numbering scheme of (**a**) the cation with interacting water molecule and (**b**) anion in T:OHN salt. Thermal displacement ellipsoids are drawn with 30% probability. Hydrogen bonds are depicted as dashed lines. (**c**) Overlay of cations in T:HCl (green) and T:OHN crystals.

Both salts, T:HCl and newly obtained T:OHN, crystalize in the monoclinic system in space symmetry groups *P*2_1_/*c* and *P*2_1_/*n*, respectively. In the case of T:OHN, except for the anion and cation pair one water molecule is present in the asymmetric part of the unit cell (Figure 3a,b). 

**Figure 4:**
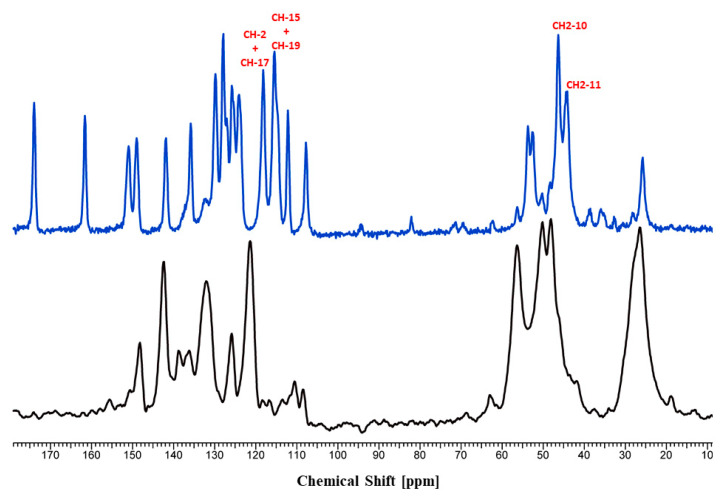
^13^C-CP/MAS-NMR spectra of T:HCl (black) and the newly obtained salt T:OHN (blue) recorded with a contact time of 4 ms. Spinning sidebands are marked with asterisks.

The comparison of the protonated trazodone molecules in both salts revealed that they are different rotamers considering on the C9-N4 bond (Figure 3c). This is reflected in the C8-C9-N4-C10 torsion angle values equal to 176.11 and −61.66° for T:HCl and T:OHN, respectively. Interestingly, on the ^13^C solid-state NMR spectra (Supplementary Materials Table S1) the difference in chemical shifts for this atom is 2.79 ppm and it is not the biggest one observed. The highest chemical shift differences are present for C15 (−6.81 ppm) and C19 (−4.51 ppm) atoms constituting the chlorobenzene ring, C10 (3.95 ppm) and C11 (4.10 ppm) from the piperazine ring, and C2 (3.44 ppm) of the fused pyridine ring (Table S1 and Figure 4). It should be underlined that the calculations of the NMR parameters (the GIPAW DFT method [27,28,29]) can be particularly helpful in assignments of the signals, as well as may provide a good way to study the dependence of NMR parameters upon structure. The calculated NMR chemical shifts of ^13^C atoms of T:HCl and T:OHN were compared with the experimental data to support the NMR spectral assignments (the CASTEP program [30] has been used to calculate carbon atoms chemical shifts and the results are presented in Table S1). It should be emphasized that the theoretical carbon atom chemical shifts are in excellent agreement with the experimental chemical shifts (Supplementary Materials Figures S1 and S2), which proves the correctness of the assignments made.

Previous studies showed that even such small differences in chemical shifts of chemically identical moieties may be attributed to the presence of weak intermolecular interactions, i.e., various hydrogen bonds or aromatic interactions [31,32,33] In the analyzed structures, the ”classical” (O–H and N–H donors) and weaker (C–H donors) hydrogen bonds are found in both crystals (Tables S2 and S3). The protonated trazodone molecule in T:OHN is interacting with the water molecule and forming the N–H···O hydrogen bond (Figure 3a). Further, the water molecule acts as a double hydrogen bond donor to both oxygen atoms of the carboxylate group of the anion. In effect, an infinite supramolecular chain structure along [101] direction is observed (Figure S4). Taking into account only O–H···O hydrogen bonds, this entity can be described with the second level graph set as *C*^2^_2_(6) [34] and the *p*1*c*1 rod symmetry group [35]. Therefore, the trazodone cations “decorate” these chains, bonding through N–H···O hydrogen bonds to water molecules. Whereas, in T:HCl, protonated piperazine interacts with chloride anion forming a definite motif. There are no further strong hydrogen bond donors, though building up 3D crystal structure occurs via weak interactions only (Figure S5).

**Figure 5:**
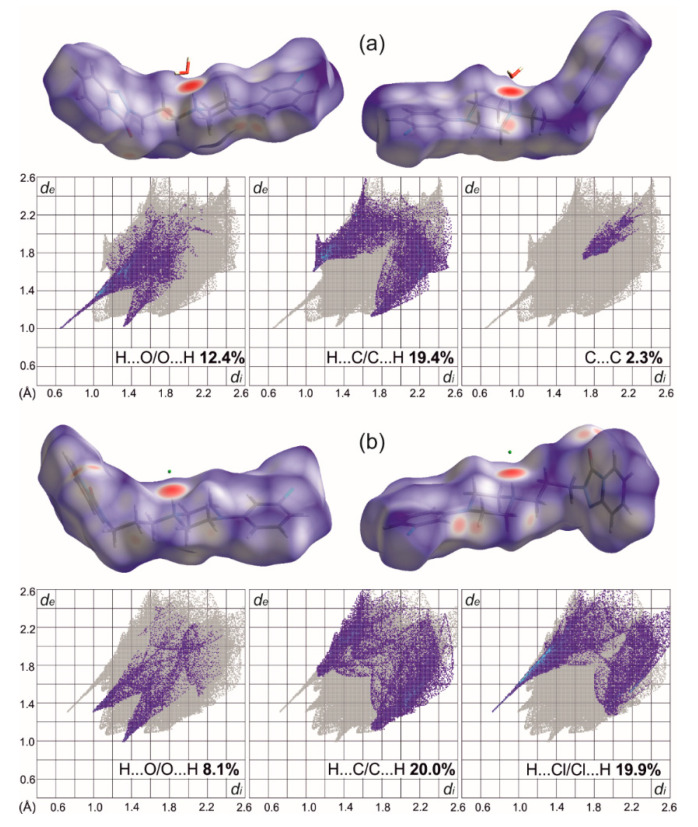
Hirshfeld surfaces (front and rear views) and decomposed fingerprint plots showing the most important interactions in T:OHN (**a**) and T:HCl (**b**).

To elucidate the weak intermolecular interactions, the most convenient method is to compare the molecular Hirshfeld surfaces [36] (Figure 5). They can be represented by surfaces normalized with VdW radii as well as so-called resolved fingerprint plots to show the types of interacting atoms [37]. On both surfaces calculated for cations and depicted with the same scaling, big red spots correspond to N–H···O(water) or N–H···Cl interactions. In the case of T:OHN pale red spots on the surface reflect weak C–H···O interactions, most of them being charge assisted [38], similarly to C–H···Cl interactions in T:HCl, also visible on the surface together with reasonably short C–H···O contacts. The resolved fingerprint plots showed also a presence of weak C–H···p interactions as well as stacking interactions in T:OHN. This is not surprising, since the anion is composed of two fused six-membered rings that align almost parallelly with the trazodone fused ring system and nearly perpendicularly to the chlorophenyl ring (see Figure S3 in the Supplementary Materials). These interactions are rank as strong ones according to calculations with the Aromatic Interactions module in the Mercury program [25] and thus might be responsible for the observed carbon atoms shielding in the area of the fused ring system and deshielding in the area of chlorophenyl ring in T:OHN. We may also conjecture that C–H···Cl and C–H···O interactions with donors from piperazine ring are somewhat stronger in T:HCl than C–H···O and C–H···π contacts in T:OHN (Tables S2 and S3). What is more, in T:HCl the strongest C–H···O hydrogen bonds join cations into a 1D chain along *2*_1_ screw axis and the anions resides in the middle of the cross-section of these chains. Hence, N–H···Cl and C–H···Cl interactions join these chains into a 3D structure. In T:OHN chains formed by strong O–H···O H-bonds between anion and water molecules are further organized into layers mostly by C–H···π and π···π interactions between anion and chlorophenyl ring of trazodone (Figure S4).

## 3. Materials and Methods

### 3.1. Materials/Synthesis

#### 3.1.1. Synthesis of T:HBr

##### Method I

In a beaker, 0.372 g of trazodone free base was dissolved in 40 cm^3^ of acetone by heating the solution to boiling. Concentrated aqueous HBr solution was then added portion wise until acidic. The solution was then cooled to 4 °C and kept under these conditions for 24 h. After this time, the resulting amorphous precipitate was filtered off. The crude product was crystallized from ethanol. Y = 46%.

##### Method II

In a beaker, 0.372 g of trazodone free base was dissolved in 20 cm^3^ of methanol by heating the solution to boiling. Concentrated aqueous HBr solution was then added portionwise until acidic. The solution was allowed to evaporate the solvent. The crude product was crystallized from ethanol. Y = 86%.

T:HBr. UPLC-MS, t_M_ = 3.91 min. Elemental analysis, (C_19_H_23_BrClN_5_O) %C = 29.31, %H = 3.86, %N = 8.31. ^1^H-NMR (300 MHz, CDCl_3_), δ (ppm): 7.75 (d, *J* = 7.1 Hz, 1H, ArH), 7.21 (d, *J* = 8.1 Hz, 1H, ArH), 7.18–7.06 (m, 2H, ArH), 6.97 (d, *J* = 7.3 Hz, 2H, ArH), 6.84 (d, *J* = 8.9 Hz, 1H, ArH), 6.58–6.50 (m, 1H, ArH), 4.17 (t, *J* = 6.0 Hz, 2H, CONCH), 3.88 (t, *J* = 12.0 Hz, 2H, CH_Pip_), 3.66 (s, 4H, CH_Pip_), 3.21 (dd, *J* = 16.6, 5.2 Hz, 2H, CH_Pip_), 3.08 (d, *J* = 12.9 Hz, 2H, NCH_Alif_), 2.59 (d, *J* = 16.8 Hz, 2H, CH_Alif_). FT-IR (cm^−1^), 2983 (C–H), 2937; 2860 (C–H), 1694 (C=O), 1640 (C=N), 1594; 1491 (C=C), 1356 (C–N), 742 (C–Cl).

#### 3.1.2. Synthesis of T:OHN

##### Method I

A solution of 0.190 g of 1-hydroxy-2-naphthoic acid in 15 cm^3^ of methanol was prepared. In a beaker, 0.372 g of trazodone free base was dissolved in 20 cm^3^ of methanol by heating the solution to reflux. The 1-hydroxy-2-naphthoic acid solution was then added portionwise while stirring. The solution was cooled to 4 °C and allowed to crystallize. The resulting crystals were filtered off. Y = 57%.

##### Method II

A solution of 0.190 g of 1-hydroxy-2-naphthoic acid in 20 cm^3^ of acetone was prepared. In a beaker, 0.372 g of trazodone free base was dissolved in 20 cm^3^ of acetone by heating the solution to boiling. The 1-hydroxy-2-naphthoic acid solution was then added portionwise to the trazodone solution with continued stirring. The solution was cooled to 4 °C and allowed to crystallize. The resulting crystals were filtered off. Y = 39%.

##### Method III

A solution of 0.190 g of 1-hydroxy-2-naphthoic acid in 30 cm^3^ of ethyl acetate is prepared. In a beaker, 0.372 g of trazodone free base was dissolved in 30 cm^3^ of ethyl acetate by heating the solution to boiling. The 1-hydroxy-2-naphthoic acid solution was then added portion-wise to the trazodone solution with continued stirring. The solution was cooled to 4 °C and allowed to crystallize. The resulting crystals were filtered off. Y = 31%.

T:OHN. UPLC-MS, t_M_ = 3.89 min. Elemental analysis, (C_30_H_30_ClN_5_O_4_) %C = 62.14, %H = 5.53, %N = 12.05. ^1^H-NMR (300 MHz, CDCl_3_), δ (ppm): 8.37 (d, *J* = 8.3 Hz, 1H, ArH), 7.83 (d, *J* = 8.7 Hz, 1H, ArH), 7.80–7.67 (m, 2H, ArH), 7.59–7.43 (m, 2H, ArH), 7.18 (dd, *J* = 18.4, 10.2 Hz, 2H, ArH), 7.04–6.93 (m, 2H, ArH), 6.85 (t, *J* = 4.0 Hz, 2H, ArH), 6.80–6.73 (m, 1H, ArH), 6.45 (ddd, *J* = 7.3, 4.9, 2.5 Hz, 1H, ArH), 4.12 (t, *J* = 6.3 Hz, 2H, CONCH), 3.48–3.30 (m, 4H, CH_Pip_), 3.13–3.01 (m, 4H, CH_Pip_), 3.02–2.89 (m, 2H, NCH_Alif_), 2.40–2.29 (m, 2H, CH_Alif_), FT-IR (cm^−1^), 3456 (O–H), 3042 (C–H Ar), 2942; 2843 (C–H), 1716 (C=O), 1638 (C=N), 1592; 1488 (C=C), 1375; 1262 (C–O), 1346 (C–N), 749 (C–Cl).

#### 3.1.3. Synthesis of T:EM

##### Method I

A solution of 0.388 g of embonic acid in 20 cm^3^ of 0.1 M NaOH was prepared. In a beaker, 0.372 g of trazodone free base was dissolved in 20 cm^3^ of 0.1M HCl. The embonic acid solution was added portionwise to the trazodone solution while stirring. The resulting mixture was adjusted to pH = 7.0. After cooling to 4 °C, a precipitate formed which was filtered off. Y = 84%.

##### Method II

A solution of 0.388 g of embonic acid in 5 cm^3^ of 1M NaOH was prepared at room temperature. In a beaker, 0.372 g of trazodone free base was dissolved in 5 cm^3^ of 1M HCl. The embonic acid solution was added portionwise to the trazodone solution while stirring. The resulting mixture was adjusted to pH = 7.0. After cooling to 4 °C, the resulting precipitate was filtered off. Y = 63%.

T:EM. UPLC-MS t_M_ 3.87 min. Elemental analysis, %C = 61.28, %H = 4.39, %N = 3.19).^1^H-NMR (300 MHz, DMSO) δ 8.39 (s, 2H, ArH), 8.15 (d, *J* = 8.6 Hz, 2H, ArH), 7.84 (dd, *J* = 15.4, 7.5 Hz, 3H, ArH), 7.34–7.12 (m, 7H, ArH), 7.03 (s, 1H, ArH), 6.95 (d, *J* = 8.3 Hz, 1H, ArH), 6.85 (d, *J* = 7.7 Hz, 1H, ArH), 6.66–6.58 (m, 1H, ArH), 4.76 (s, 4H, CH_Alif-Pam_), 4.02 (dd, *J* = 12.2, 6.4 Hz, 2H, CONCH), 3.17 (dd, *J* = 20.7, 11.3 Hz, 8H, CH_Pip_), 2.18 (d, *J* = 6.5 Hz, 2H, NCH_Alif_), 1.30 (dd, *J* = 14.6, 7.4 Hz, 2H, CH_Alif_). FT-IR (cm^−1^), 3280 (O–H), 2970 (C–H Ar), 2949; 2840 (C–H), 1657(C=O), 1633 (C=N), 1594; 1453 (C=C), 1353; 1205 (C–O), 1339 (C–N), 738 (C–Cl).

### 3.2. Powder X-ray Diffraction (PXRD)

Powder X-ray diffraction patterns for a bulk sample of T:OHN (after recrystallization) were recorded at room temperature on a Bruker Advance D8 diffractometer (Bruker, Billerica, MA, USA) equipped with a LYNXEYE position sensitive detector using CuKα radiation (λ = 0.15406 nm). The data were collected in the Bragg−Brentano (θ/θ) horizontal geometry (flat reflection mode) between 4° and 50° (2θ) in a continuous scan using 0.03° steps and 384 s/step. The diffractometer incident beam path was equipped with a 2.5° Soller slit and a 1.14° fixed divergence slit, while the diffracted beam path was equipped with a programmable anti-scatter slit (fixed at 2.20°), a Ni β-filter, and a 2.5° Soller slit. The diffraction pattern is presented in Figure S3 in the Supplementary Materials file and compared to the pattern simulated from single crystal final refinment data. These results confirm sample purity and homogeneity.

### 3.3. Single Crystal X-ray Diffraction (SCXRD)

X-ray reflections for T:OHN were measured at room temperature on a Rigaku Oxford Diffraction Gemini A Ultra diffractometer (Rigaku Corporation, Tokyo, Japan) using mirror monochromated CuKα radiation (λ = 1.54184 Å). Data collection, cell refinement, and data reduction were performed with CrysAlisPro software (CrysAlisPro 1.171.39.46, Rigaku OD, 2018). The empirical absorption corrections using spherical harmonics, implemented in the multiscan algorithm were performed. The structure was solved with the SHELXT [39] structure solution program using Intrinsic Phasing and refined with the SHELXL refinement package [40] using Least Squares minimization. implemented in the OLEX2 v.1.3 suite [41]. All non-hydrogen atoms were refined anisotropically. H atoms on C atoms were added to the structure model at geometrically idealized positions and refined as riding atoms, with Uiso(H) = 1.2 × Ueq(CH) and 1.5U × eq(CH_3_). H atoms of hydroxy and amino groups were located from a Fourier map and refined with a fixed isotropic parameter of the parent atom equal to 1.2 × Ueq and 1.5U × eq for NH and OH, respectively. Water molecule was refined as a rigid group (AFIX 6). Molecular diagrams were generated using ORTEP-3 for Windows [26] (Figure 3), Diamond [42] (Figure S4), and Mercury 2.0 [25]. (Figures S5 and S6) programs whereas geometrical parameters were calculated using OLEX2 [41] and Platon package [43,44]. Crystal Data for T:OHN: C_30_H_32_ClN_5_O_5_: (*M* =578.05 g/mol): monoclinic, space group *P*2_1_/*n* (no. 14), *a* = 8.17642(17) Å, *b* = 36.3453(5) Å, *c* = 10.12988(19) Å, *β* = 109.651(2)°, *V* = 2835.01(10) Å^3^, *Z* = 4, T = 298.15 K, μ(Cu Kα) = 1.600 mm^−1^, *Dcalc* = 1.354 g/cm^3^, 60220 reflections measured (9.586° ≤ 2Θ ≤ 134.566°), 5065 unique (*R*_int_ = 0.0768, *R*_sigma_ = 0.0254) which were used in all calculations. The final *R*_1_ was 0.0438 (I > 2σ(I)) and *wR_2_* was 0.1163 (all data). CCDC deposition number: 2051183.

### 3.4. Solid-State Nuclear Magnetic Resonance (SSNMR)

The solid-state ^13^C-NMR spectra were obtained on a Bruker Avance 400 WB (Bruker, Rheinstetten, Germany) utilizing a ^13^C resonant frequency of 100.61 MHz (magnetic field strength of 9.39 T). Approximately 100 mg of crystalline sample was packed into a zirconium rotor with a Kel-F cap. The cross‑polarization, magic angle spinning (CP-MAS) pulse sequence was used for spectral acquisition. Each sample was spun at a frequency of 8 kHz, and the magic angle setting was calibrated by the KBr method. Each data set was subjected to an 8 Hz line broadening factor and subsequently, Fourier transformed and phase corrected to produce a frequency domain spectrum. The chemical shifts were referenced to TMS using adamantane as an external secondary standard. The optimized recycle delay for T:HCl and T:OHN was 170 s and 50 s, respectively. The NMR spectra were processed with the ACD/SpecManager NMR program (version 10.0, Advanced Chemistry Development, Inc., Toronto, ON, Canada).

### 3.5. GIPAW DFT Calculations

The quantum-chemical calculations of geometry optimization and NMR shielding constants were carried out with the CASTEP [28,29] program implemented in the Materials Studio 6.1 software (Accelrys Software Inc.: San Diego, CA, USA. 2013) using the plane-wave pseudopotential formalism and the Perdew-Burke-Ernzerhof (PBE) [45] exchange-correlation functional, defined within the generalized gradient approximation (GGA) [46], the calculations were done with ultrasoft pseudopotentials calculated on the fly. The quality of calculations was set to fine as implemented in the CASTEP standards. CASTEP default values for the geometry convergence criteria were used. The kinetic energy cutoff for the plane waves was set to 550 eV. Brillouin zone integration was performed using a discrete 2 × 2 × 1 for Monkhorst−Pack k-point sampling for a primitive cell. The computation of shielding tensors was performed using the Gauge Including Projector Augmented Wave Density Functional Theory (GIPAW) method of Pickard et al. In the calculations the experimental X-ray structures were used, but the positions of all atoms were optimized, while the cell parameters were fixed. To compare the theoretical and experimental data, the calculated chemical shielding constants (σ_iso_) were converted to chemical shifts (δ_iso_), using the following equation: δ_iso_ = (σ_Gly_ + δ_Gly_) − σ_iso_, where σ_Gly_ and δ_Gly_ stand for the shielding constant and the experimental chemical shift, respectively, of the glycine carbonyl carbon atom (176.50 ppm).

### 3.6. Preparation of Tablets

Cylindrical tablets were prepared by direct compression of the appropriate trazodone compound with lactose monohydrate (lactose monohydrate; LA, p.a. grade). All tablets were prepared using a laboratory press fitted with a 14 mm flat-faced punch and die set, applying 5 t force for 30 s. Each tablet contained 150 mg lactose monohydrate and an appropriate trazodone compound equivalent to 75 mg of trazodone (the amount corresponding to market preparations, e.g., Trittico). The amount of the active substance in the tablet: 75 mg ± 5%. As a reference tablets with T:HCl + LA were used.

### 3.7. In Vitro Dissolution Profile Studies

Dissolution experiments were carried out using Erweka DT-80 (Erweka GmbH., Heusenstam, Germany) equipment with controlled temperature (at 36 ± 1 °C) for 4 h. In this method, hydrochloric acid solution (concentration 0.01M) was used as dissolution medium. The rate of stirring for the whole experiment time was 50 ± 2 rpm. In all formulations, the amount of trazodone was 75 mg. Prepared tablets were placed in 900 cm^3^ of acceptor fluid. For 240 min at appropriate intervals, 5 cm^3^ of the sample were taken. Samples were taken after 1, 2, 5, 10, 15, 30, 45, 60, 90, 120, 150, 180, 210, and 240 min. To maintain the constant volume of the dissolution medium after each sampling the fresh portion of the medium was added. All samples were filtered with cellulose syringe filters with a membrane diameter of 0.45 µm Millipore filter and diluted.

**Figure 6:**
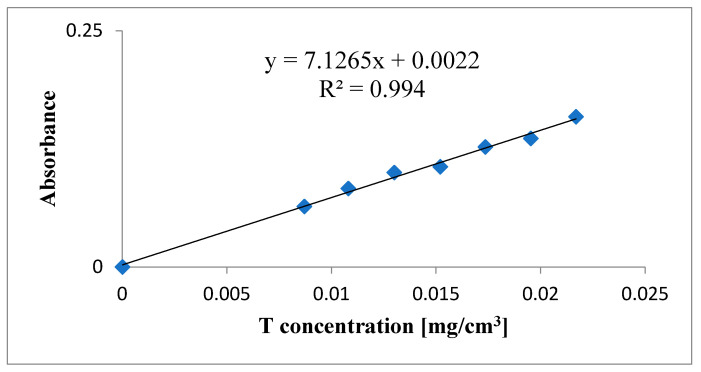
Calibration curve.

**Figure 7:**
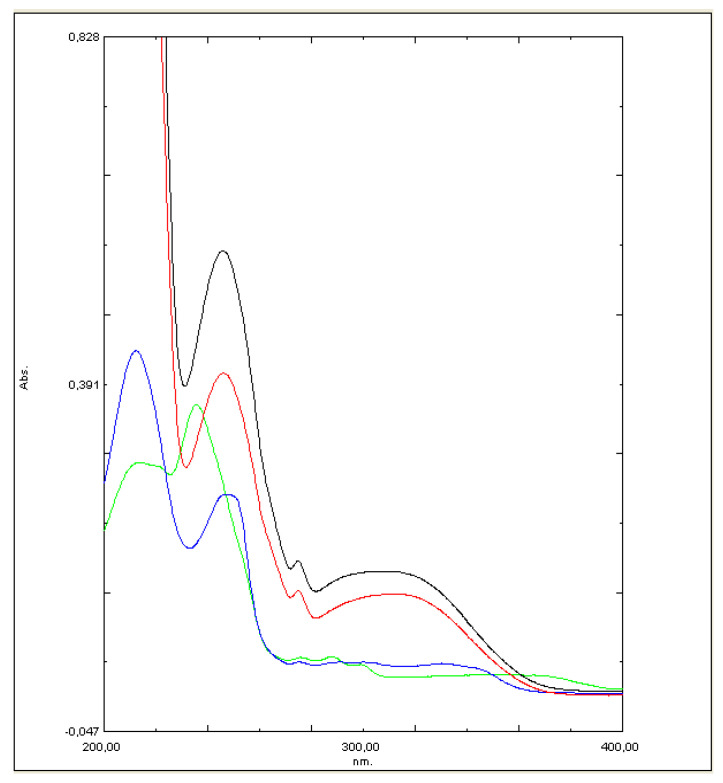
Absorbance curve for the tested compounds: (black T:HCl; red T:HBr; blue T:OHN; green T:EM).

Shimadzu UV-1800 UV-Vis spectrophotometer (Shimadzu Co., Kyoto, Japan) was used to analyze all samples. To minimize possible errors because of overlapping peaks, the maximum in trazodone UV-Vis spectrum at 311 nm was used to calculate drug concentrations without any interferences. The calibration curve used in these studies was presented in Figure 6. At 311 nm wavelength, a saturated solution of lactose monohydrate has only negligible absorbance (~0.001). To calculate the drug release from each of the formulations based on the separately constructed calibration curve (R^2^ > 0.994) the mean of 3 determinations was used (Figure 7).

UPLC-MS analysis conditions: Waters UPLC with PDA detector. Column: 1.7 μm Aquity UPLC BEH C18 (Waters Corporation, Milford, MA, USA). Mobile phase: methanol: water + formic acid (4:6 + 0.1%, *v/v*). Ionization method: electrospray. Detector: quadrupole. Mass spectra were recorded with positive mode. ^1^H-NMR: Bruker 400 MHz instrument (Bruker, Rheinstetten, Germany) with TMS (trimethylsilane) as the internal standard.

## 4. Conclusions

The search for simple and at the same time economically and ecologically beneficial methods of synthesis of complex chemical molecules is currently the main goal of modern organic synthesis. In this work, a new method of trazodone synthesis under microwave irradiation was used, and then two new trazodone salts, T:HBr and T:OHN, were obtained in two (or three) different ways. To determine their therapeutic usefulness, we evaluated the dissolution profiles of trazodone from tablets containing tested salts with added lactose monohydrate. It turned out that the trazodone salt T:HBr showed a much faster release time of the active substance compared to the reference T:HCl salt. On the other hand, T:OHN showed a slow-release profile of the active substance concerning the commercially available hydrochloric salt. Changing the dissolution profile of the drug (indicating slower release) may be useful when designing new formulations containing this drug. Based on these preliminary studies, it may be tempted to state that the salts of trazodone with small anions (T:HCl, T:HBr), due to only weak interactions occurring in the crystal lattice, are released from the tablet faster than salts containing much larger anions (T:OHN, T:EM). Spacious anions, especially those with aromatic substituents or H-bond active groups, create more significant interactions with the cation, making them more difficult to break, which could explain the retarded release time of the drug.

## Figures and Tables

**Table 1:** Tablet disintegration time.

Tablet Composition	Tablet Disintegration Time
T:HCl + LA	13.5 min
T:HBr + LA	5.5 min
T:OHN + LA	more than 24 h: after this time, approximately 100% of the API was released from tablets with LA
T:EM + LA	more than 24 h: after this time, approximately 90% of the API was released from the tablet with LA

## Supplementary Materials

The following are available online. Figure S1: Comparison of experimental and teoretical chemical shifts of carbon-13 of T:HCl, Figure S2. Comparison of experimental and teoretical chemical shifts of carbon-13 of T:OHN, Figure S3. Experimental powder X-ray diffraction pattern for bulk T:OHN sample (upper chart) and simulated from single crystal data (lower chart), Figure S4. Weak interactions motives found in T:OHN (a) and T:HCl (b) crystals, Figure S5. Packing diagram for T:OHN crystal. View along [101] direction, Figure S6. Packing diagram for T:HCl crystal. View along [010] direction Table S1: ^13^C CP/MAS NMR chemical shifts (ppm) of T:HCl and T:OHN., Table S2. Geometry of main weak intermolecular interactions in T:OHN crystal, Table S3 Geometry of main weak intermolecular interactions in T:HCl crystal.

## Sample Availability

Samples of compounds T:HBr and T:OHN are available from the authors.
