# Supplementary material for: New Pharmaceutical Salts of Trazodone
Source: Molecules. 2021 Feb 2;26(3):769. doi: 10.3390/molecules26030769 (PMC7867375; doi:10.3390/molecules26030769)
Supplement: Supplementary file 1 [file molecules-26-00769-s001.pdf]

molecules

MDPI

SUPPORTING INFORMATION

NEW PHARMACEUTICAL SALTS OF TRAZODONE

Jolanta Jaśkowska [1,*], Przemysław Zaręba [1], Anna Drabczyk [1], Agnieszka Kozak [2], Izabela D. Madura [3], Zbigniew Majka [4] and Edyta Pindelska [2,*]

[1] Institute of Organic Chemistry and Technology, Faculty of Chemical and Engineering and Technology, Cracow University of Technology, 24 Warszawska Street, 31-155 Cracow, Poland; przemyslaw.zareba@pk.edu.pl (P.Z.); anna.drabczyk@pk.edu.pl (A.D.).

[2] Department of Analytical Chemistry and Biomaterials, Faculty of Pharmacy, Medical University of Warsaw, Banacha 1, 02-093 Warsaw, Poland; asokal@wum.edu.pl.

[3] Faculty of Chemistry, Warsaw University of Technology, Noakowskiego 3, 00-664 Warsaw, Poland; izabela@ch.pw.edu.pl.

[4] Zbigniew Majka Consulting, ul Górczewska 200c /58, 01-460 Warszawa, Poland; zbig_majka@wp.pl.

* Correspondences: jolanta.jaskowska@pk.edu.pl (J.J.); edyta.pindelska@wum.edu.pl (E.P.); Tel.: +48-12-628-27-40 (J.J.); Tel.: +48-22-572-07-57 (E.P.)

Contents:

Page 2: Table S1. ^{13}C CP/MAS NMR chemical shifts (ppm) of T:HCl and T:OHN.

Page 2: Figure S1. Comparison of experimental and theoretical chemical shifts of carbon-13 of **T:HCl**.

Page 3: Figure S2. Comparison of experimental and theoretical chemical shifts of carbon-13 of **T:OHN**.

Page 3: Figure S3. Experimental powder X-ray diffraction pattern for bulk **T:OHN** sample (upper chart) and simulated from single crystal data (lower chart).

Page 4: Table. S2. Geometry of main weak intermolecular interactions in **T:OHN** crystal.

Page 4: Table. S3. Geometry of main weak intermolecular interactions in **T:HCl** crystal.

Page 4: Figure S4. Weak interactions motives found in **T:OHN** (a) and **T:HCl** (b) crystals.

Page 5: Figure S5. Packing diagram for **T:OHN** crystal. View along [101] direction.

Page 5: Figure S6. Packing diagram for **T:HCl** crystal. View along [010] direction.

Table S1. ^{13}C CP/MAS NMR chemical shifts (ppm) of T:HCl and T:OHN.

Group	Assignment	T:HCl δ [ppm]		T:OHN δ [ppm]		T:HCl δexp - T:OHN δexp [ppm]
		EXP	CAL*	EXP	CAL*	
**C**	**1**	148,37	146,19	149,02	147,56	-0,65
CH	2	121,62	118,35	118,18	117,39	3,44
CH	3	132,32	132,57	129,83	130,09	2,49
CH	4	108,63	111,25	107,76	109,85	0,87
CH	5	126,08	125,74	125,85	125,66	0,23
C	6	142,64	142,05	141,89	142,06	0,75
CH_2	7	48,26	44,6	46,35	42,25	1,91
CH_2	8	26,4	19,8	25,79	22,76	0,61
CH_2	9	56,54	50,87	53,75	50,35	2,79
CH_2	10	50,3	49,79	46,35	41,83	3,95
CH_2	11	48,26	41,62	44,16	40,02	4,1
CH_2	12	48,26	44,34	46,35	42	1,91
CH_2	13	50,3	46,14	52,66	49,09	-2,36
C	14	148,37	150,1	150,94	149,54	-2,57
CH	15	108,63	111,71	115,44	112,48	-6,81
CH	16	131,3	131,05	129,83	129,57	1,47
CH	17	121,24	116,77	118,18	117,38	3,06
C	18	142,64	142,01	141,89	139,97	0,75
CH	19	110,93	112,89	115,44	115,15	-4,51
C	20			112,15	110,25	
C	21			161,63	165,95	
C	22			161,63	165,95	
CH	23			127,91	126,62	
CH	24			124,07	125,53	
CH	25			127,91	128,98	
CH	26			129,83	130,38	
C	27			135,86	134,87	
CH	28			141,89	141,58	
CH	29			129,83	129,13	
C	30			173,97	175,35	

*CASTEP computed δ values have been obtained from all atoms positions optimization (the lattice parameters were fixed to their experimental values);.

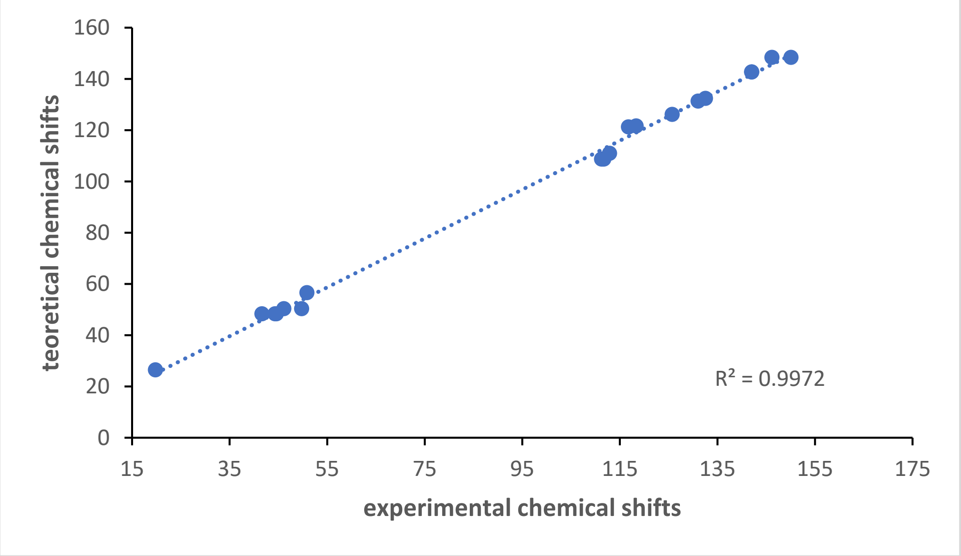

Figure S1. Comparison of experimental and theoretical chemical shifts of carbon-13 of **T:HCl**.

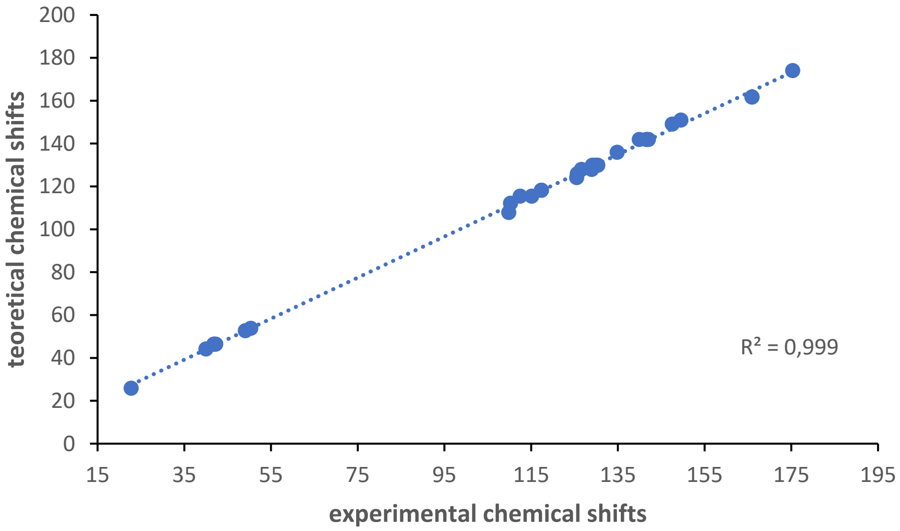

Figure S2. Comparison of experimental and teoretical chemical shifts of carbon-13 of **T:OHN**.

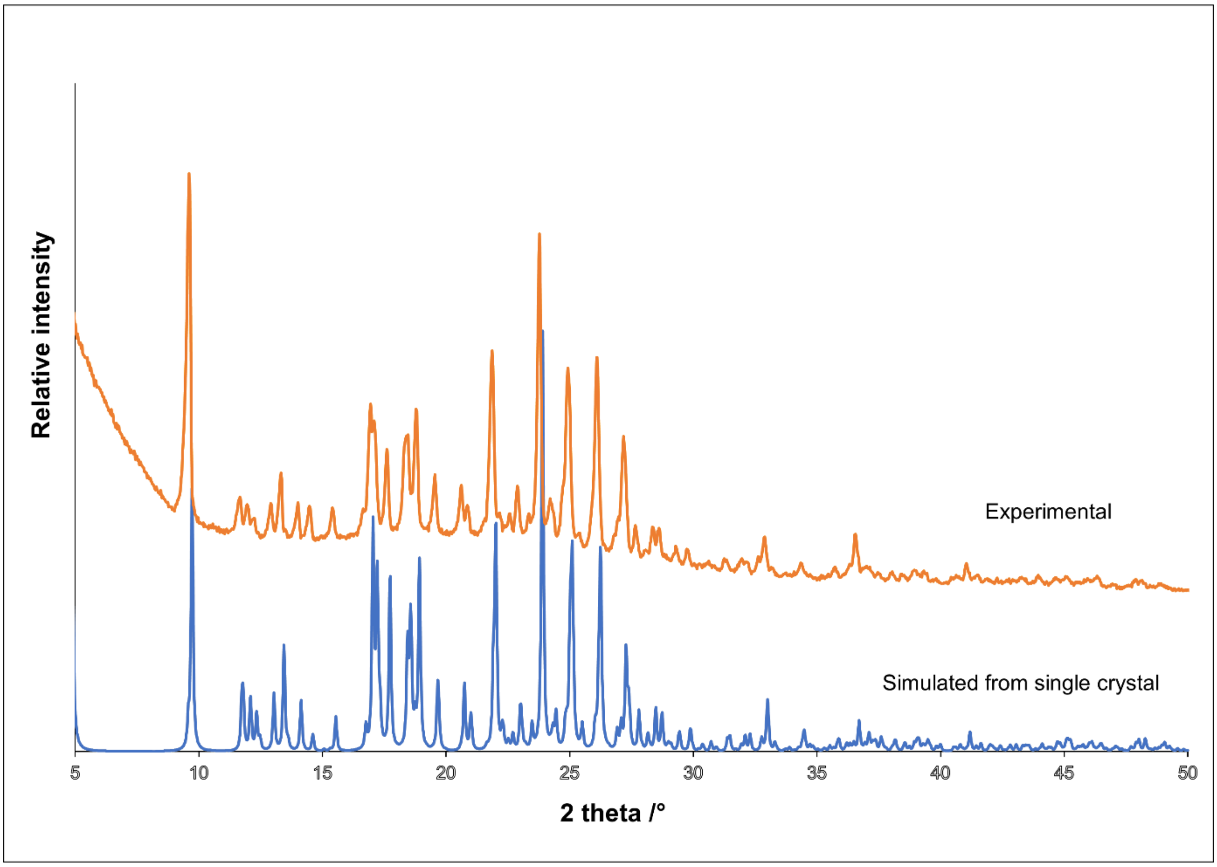

Figure S3. Experimental powder X-ray diffraction pattern for bulk **T:OHN** sample (upper chart) and simulated from single crystal data (lower chart).

Table S2. Geometry of main weak intermolecular interactions in **T:OHN** crystal.

Caption	d(H-A)/Å	d(D-A)/Å	D-H-A/°
N4–H4…O5	1.72(2)	2.651(2)	169.4(18)
O4–H4B…O3	1.50(3)	2.457(2)	159(3)
O5–H5A…O2^{i}	1.86	2.710(2)	177.1
O5–H5B…O3^{ii}	1.85	2.695(2)	176.8
C9–H9B…O2^{ii}	2.49	3.351(2)	147
C10–H10A…O1^{iii}	2.44	3.166(3)	132
C10–H10A…Cg1a^{iv}	2.91	3.327(2)	107
C11–H11B…Cg2a^{iv}	2.73	3.645(2)	158
C12–H12A…O4^{v}	2.48	3.405(2)	160
C13–H13A…O2^{ii}	2.55	3.390(2)	146
C15–H15…O4^{v}	2.54	3.454(2)	169
C30–H30…O5^{i}	2.59	3.486(3)	161
Cg(t6)…Cg(1a)^{ii}		4.184(2)	
Cg(PhCl)…Cg(PhCl^{iv})		4.766(2)	

Symmetry operations: [i] -x,1-y,1-z; [ii] 1/2-x,-1/2+y,3/2-z; [iii] -1/2+x,1/2-y,-1/2+z; [iv] 1-x,1-y,1-z; [v] 1-x,1-y,2-z. Ring codes: Cg1a, Cg2a – rings of the anion; Cg(t6) – six membered ring of the fused 9-membered ring (head); Cg(PhCl) – chlorophenyl ring.

Table S3. Geometry of main weak intermolecular interactions in **T:HCl** crystal.

Caption	d(H-A)/Å	d(D-A)/Å	D-H-A/°
N4–H49…Cl2	1.97	3.0418	173
C8–H31…Cl2^{i}	2.68	3.5870	140
C9–H50 …Cl2^{ii}	2.54	3.5833	157
C10–H40…Cl2^{iii}	2.69	3.7176	155
C11–H34…O1^{iii}	2.37	3.2058	132
C12–H35…O1^{i}	2.50	3.2541	125
C13–H36…Cl2^{ii}	2.63	3.6541	154
C19–H47…O1^{iii}	2.31	3.3964	173
Cg(t5)…Cg(t6)^{ii}		4.6241	
Cg(PhCl)…Cg(PhCl)^{iv}		4.8828	

Symmetry operations: [i] [-x, 2-y,1-z]; [ii] x,3/2-y,-1/2+z; [iii] -x,-1/2+y,3/2-z; [iv] -1-x,1-y,1-z. Ring codes: Cg(t5), Cg(t6) – five- and six membered ring of the fused 9-membered ring (head); Cg(PhCl) – chlorophenyl ring.

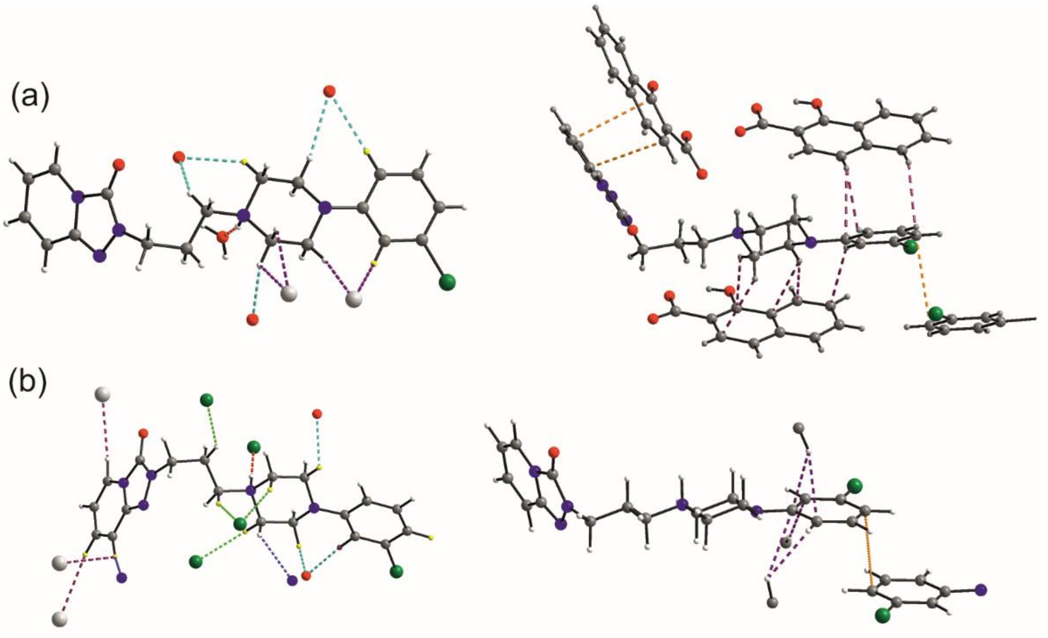

Figure S4. Weak interactions motives found in **T:OHN** (**a**) and **T:HCl** (**b**) crystals.

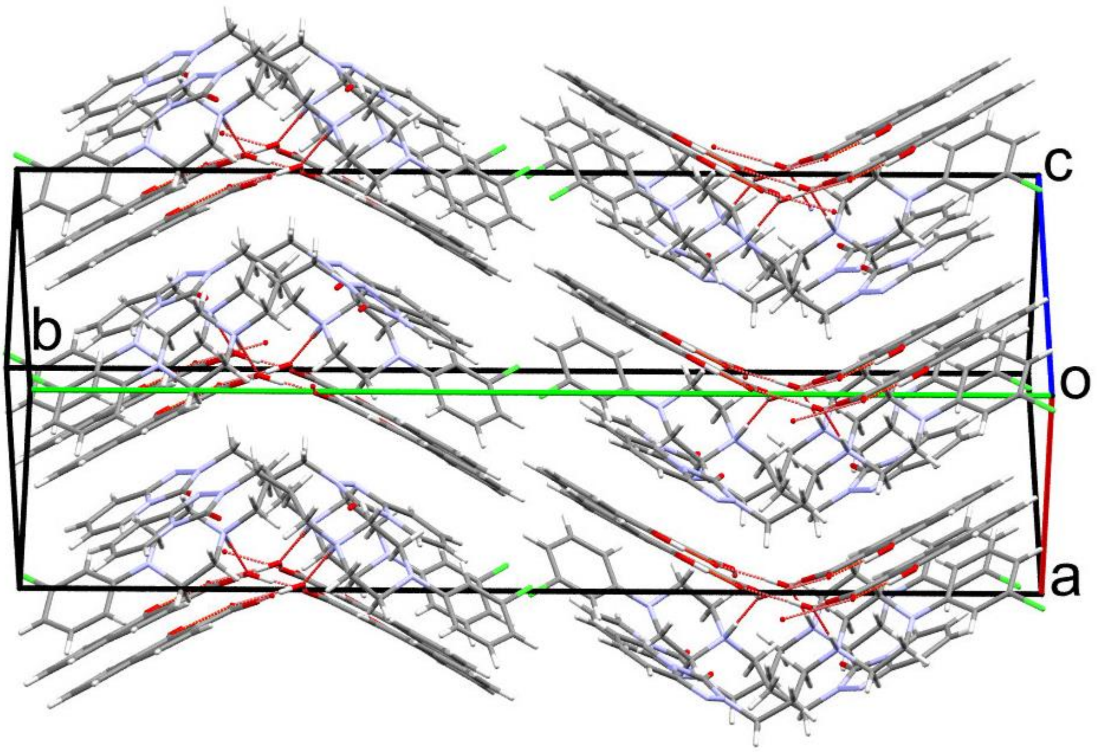

Figure S5. Packing diagram for **T:OHN** crystal. View along [101] direction.

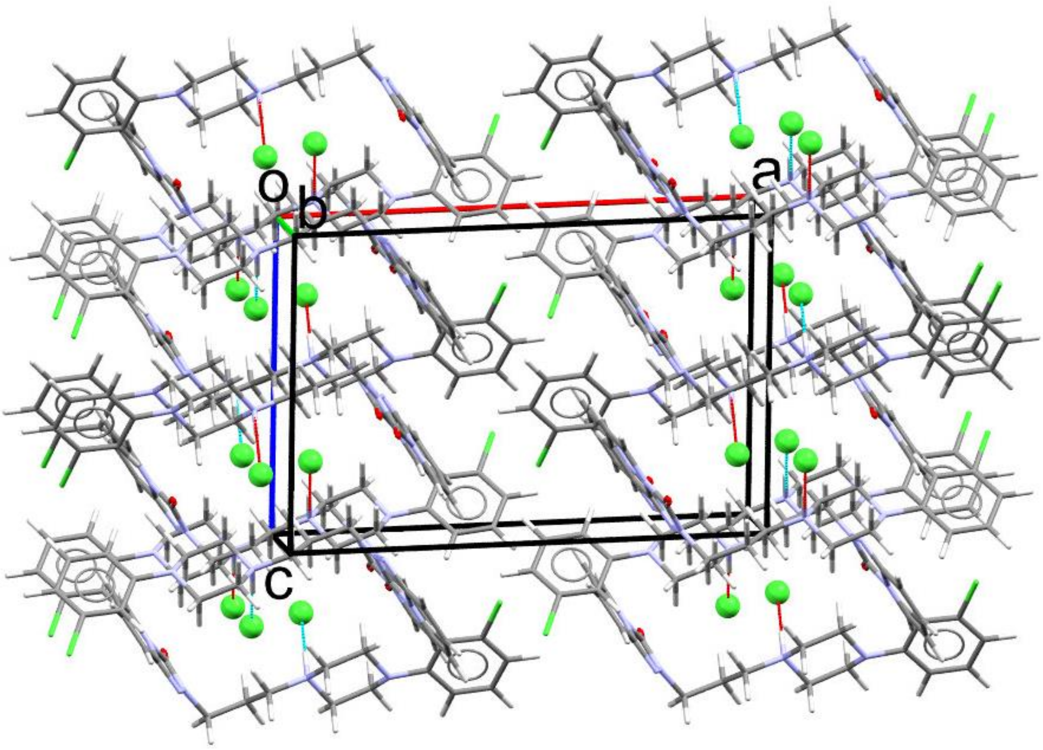

Figure S6. Packing diagram for **T:HCl** crystal. View along [010] direction.
